# Association Between Plasma Homocysteine, Folate, Vitamin B12 Levels, and Metabolic Dysfunction Indices in Elderly with Arterial Stiffness

**DOI:** 10.3390/jcm14092998

**Published:** 2025-04-26

**Authors:** Jintana Sirivarasai, Prapimporn Chattranukulchai Shantavasinkul, Manasid Thitiwiwatkul, Wutarak Monsuwan, Pachara Panpunuan, Piyamitr Sritara

**Affiliations:** 1 Nutrition Unit, Faculty of Medicine Ramathibodi Hospital, Mahidol University, Bangkok 10400, Thailand; wutarak.pue@mahidol.edu; 2 Division of Nutrition and Biochemical Medicine, Department of Medicine, Faculty of Medicine Ramathibodi Hospital, Mahidol University, Bangkok 10400, Thailand; sprapimporn@gmail.com; 3 Faculty of Medicine Ramathibodi Hospital, Mahidol University and HRH Princess Chulabhorn College of Medical Science, Bangkok 10400, Thailand; momanasid@gmail.com; 4 Division of Cardiology, Department of Medicine, Faculty of Medicine Ramathibodi Hospital, Mahidol University, Bangkok 10400, Thailand; pachpan@hotmail.com (P.P.); piyamitr.sri@mahidol.ac.th (P.S.)

**Keywords:** hyperhomocysteinemia, metabolic dysfunction indices, elderly, arterial stiffness, cardio/ankle vascular index

## Abstract

**Background**/**Objectives**: Arterial stiffness is a prevalent age-related condition that can significantly increase the risk of cardiovascular disease and mortality in older adults. Understanding the factors that contribute to vascular health, including metabolic dysfunction and hyperhomocysteinemia, alongside vitamin B status, is essential for developing effective interventions. This study aimed to explore the relationship between plasma levels of homocysteine, folate, and vitamin B12, as well as various indices of metabolic dysfunction, in elderly individuals with arterial stiffness. **Methods**: We conducted a cross-sectional analysis involving 884 participants aged 65 and older, assessing arterial stiffness using the cardio/ankle vascular index method. Additionally, we collected fasting blood samples to evaluate plasma homocysteine, folate, vitamin B12 levels, and other relevant biochemical markers. **Results**: Higher plasma homocysteine levels are significantly correlated with elevated CAVI scores and increased indices of metabolic dysfunction (*p* < 0.05). Furthermore, a multivariate logistic regression analysis demonstrated that elevated plasma homocysteine levels, along with higher levels of lipid accumulation product (LAP), triglyceride/glucose index (TyG), and visceral adiposity index (VAI), are associated with increased arterial stiffness. **Conclusions**: These findings suggest that monitoring and optimizing homocysteine, folate, and vitamin B12 levels may be beneficial for preventing or managing arterial stiffness and related metabolic disorders in the elderly population.

## 1. Introduction

Arterial stiffness, assessed through the cardio/ankle vascular index (CAVI), is a crucial biomarker for evaluating cardiovascular risk factors [1]. This measurement provides valuable insights into the elasticity of arterial walls, which can indicate the presence of underlying cardiovascular issues [1]. High levels of arterial stiffness are associated with an increased risk of many arteriosclerotic diseases, such as coronary artery disease, carotid arteriosclerosis, chronic kidney disease, and cerebrovascular disease [2]. The CAVI cut-off value, typically set at 9.0, serves as a threshold for identifying individuals at a higher risk of developing these conditions, allowing for early intervention and management strategies [1]. The elderly population is especially vulnerable to arterial stiffness due to structural and functional changes in the arterial walls, including increased collagen deposition, reduced elastin content, and vascular smooth muscle cell alterations [3]. In addition, important risk factors for arterial stiffness in older adults have been identified, including age, metabolic syndrome, sedentary lifestyle, smoking, alcohol consumption, and diet [3]. Findings from a multicenter prospective longitudinal study showed that the CAVI significantly predicted cardiovascular morbimortality and all-cause mortality after adjustment for CV risk factors, in particular for subjects ≥60 years [4].

Dietary factors play a crucial role in influencing arterial stiffness; diets high in fruits, vegetables, whole grains, and omega-3 fatty acids have been associated with improved vascular health, while the excessive intake of saturated fats, transfats, and sugar can exacerbate stiffness [5]. Furthermore, vitamin B deficiency, particularly in vitamin B9 and vitamin B12, emerges as a significant factor in hyperhomocysteinemia, which is characterized by elevated levels of homocysteine in the blood (greater than 15 µmol/L) [5]. Elevated levels of homocysteine in the blood can lead to endothelial dysfunction, promote oxidative stress, and induce inflammation, all of which contribute to structural changes in the arterial walls. This can result in the reduced elasticity and increased stiffness of the arteries [6]. Hyperhomocysteinemia is primarily influenced by deficiencies in vitamin B6, B12, and folate, which are crucial for the metabolism of homocysteine. The enzymes involved include methylenetetrahydrofolate reductase (MTHFR), which converts 5,10-methylenetetrahydrofolate to 5-methyltetrahydrofolate, facilitating the remethylation of homocysteine to methionine via methionine synthase (MS), dependent on vitamin B12 [6]. Additionally, cystathionine β-synthase (CBS) plays a role in the transsulfuration pathway, converting homocysteine to cystathionine when vitamin B6 is available. Deficiencies or mutations in these metabolic pathways can lead to elevated levels of homocysteine [6]. A previous study found significant interaction effects between vitamin B12 and Hcy levels as well as sex on the risk of all-cause mortality (*p* = 0.023 and *p* = 0.031, respectively) [7].

In addition, hyperhomocysteinemia can lead to metabolic dysfunction by promoting adiposity and dyslipidemia through several mechanisms, including increased oxidative stress, endothelial dysfunction, and inflammation [8]. A previous study on aged male Wistar rats found an impact of Hcy on cardiovascular dynamics through reduced Nrf2 protein levels, but cytokines were increased and TLR2 gene expression was altered [9]. Furthermore, elevated homocysteine levels may disrupt lipid metabolism, leading to abnormal lipid profiles characterized by increased triglycerides and altered cholesterol levels [10]. This dysregulation can contribute to the accumulation of fat tissue, particularly visceral fat, which is associated with a higher risk of metabolic syndrome, insulin resistance, and cardiovascular diseases [10]. There were significant associations of homocysteine status and homocysteine metabolism enzyme polymorphisms with hypertension and dyslipidemia (hypertriglyceridemia) in a Chinese hypertensive population (N = 228, mean age of 65.53 years) [11]. Hyperhomocysteinemia was significantly associated with an increased risk of high total cholesterol and LDL cholesterol (*p* < 0.05), together with an elevated risk of dyslipidemia in female subjects (OR = 1.30, 95%CI; 1.07–1.59) [12].

Based on this evidence, a plausible research hypothesis for this study could be that elevated levels of homocysteine contribute to metabolic dysfunction and increased arterial stiffness, ultimately leading to a higher risk of cardiovascular diseases among elderly populations. Previous studies tend to emphasize individual aspects, but recognizing their interactions is crucial for advancing our understanding of cardiovascular health. Investigating this relationship could provide insights into potential therapeutic targets for improving arterial health in at-risk populations.

## 2. Materials and Methods

### 2.1. Participants

**Figure 1:**
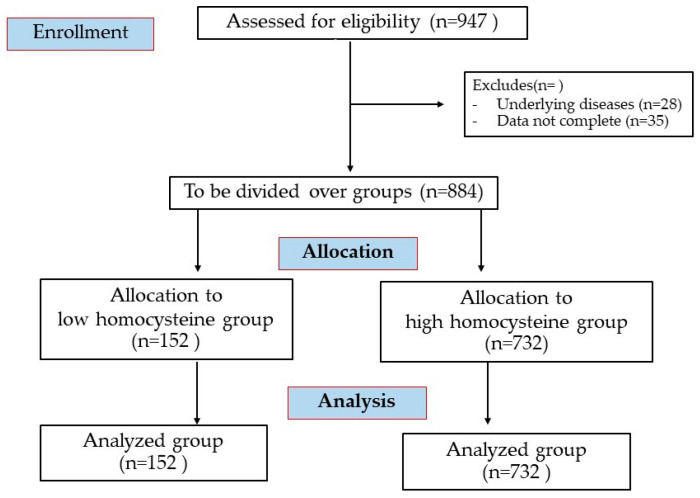
CONSORT flow diagram of this cross-sectional study.

A total of 884 participants (aged 61–85 years) were involved in the Electricity Generating Authority of Thailand (EGAT) cohort study. The initial survey began in 1985 and primarily focused on established cardiovascular disease (CVD) risk factors, with follow-up surveys conducted every five years. In this study, we utilized data from the EGAT 2012, which included comprehensive information on participants’ characteristics and health status (The details of CONSORT flow diagram and strobe statement are described in Figure 1 and Supplementary Table S1). These data were collected through structured questionnaires and health examinations. The variables assessed included age, sex, educational level, occupation, tobacco use, alcohol consumption, physical activity, and medical and family history. Participants were unequivocally excluded from the study if they presented with any of the following conditions: circulatory system diseases (including coronary artery disease, peripheral vascular disease, systemic hypertension, congestive heart failure, valvular heart disease, and cardiomyopathy or left ventricular hypertrophy), vasoactive medications, pulmonary, renal, or hematological disorders, chronic inflammatory diseases, or medications known to alter plasma homocysteine levels (such as vitamin B12 or folate antagonists and corticosteroids). This approach ensures this study’s integrity and validity. This study was performed under the approval of the Ethics Committee of the Faculty of Medicine Ramathibodi Hospital, Mahidol University (COA No. MURA2024/26). Informed consent to this study was obtained by all the participants.

### 2.2. Definition and Criteria of Metabolic Syndrome

This study employed the diagnostic criteria for metabolic syndrome (MetS), as defined by the National Cholesterol Education Program Adult Treatment Panel III (NCEP ATP III). MetS is diagnosed if an individual meets three or more of the following five criteria: waist circumference exceeding 40 inches for men or 35 inches for women, fasting blood sugar levels exceeding 100 mg/dL, fasting high-density lipoprotein (HDL) cholesterol levels below 40 mg/dL for men or 50 mg/dL for women, fasting triglyceride (TG) levels exceeding 150 mg/dL, and blood pressure surpassing 130/85 mmHg [13].

### 2.3. Body Mass Index and Anthropometrics

The BMI was calculated as weight in kilograms divided by the square of height in meters. The World Health Organization recommendations for Asian populations were used to categorize individuals into four BMI groups: <18.5 kg/m^2^ (underweight), 18.5–22.9 kg/m^2^ (normal weight), 23.0–24.9 kg/m^2^ (overweight), and ≥25 kg/m^2^ (obese) [14].

The body composition assessment utilizing the Tanita BC-418 MA Analyzer, manufactured by Tanita Co., Tokyo, Japan, adhered to the prescribed protocol and was conducted under standard operating conditions. Participants were required to observe specific guidelines, including a 24 h abstinence from food and alcohol consumption prior to the test and refraining from strenuous physical activity for at least 12 h prior to undergoing the BIA analysis.

### 2.4. Biochemical Analyses

In order to prepare for blood collection, study participants were given specific instructions to fast for a period of 12 h and abstain from engaging in any form of physical exercise for 48 h. Subsequently, venous blood samples were collected during the fasting period, specifically between the hours of 8:00 AM and 10:00 AM. Fasting plasma glucose (FPG), triglycerides (TG), total cholesterol (TC), high-density lipoprotein cholesterol (HDL-C), aspartate transaminase (AST), alanine transaminase (ALT), gamma-glutamyl transpeptidase (γ-GTP), blood urea nitrogen (BUN), creatinine (Cr), and uric acid (UA) were also measured. These measurements were carried out using established methods on advanced laboratory equipment, specifically the Cobas Analyzer and the Cobas Integra Analyzer (Roche Diagnostics Ltd., Rotkreuz, Switzerland). Homocysteine, vitamin B12, and folate levels were determined using the Immulite^®^ immunoassay automated analyzer and reagents (Diagnostic Products Corporation, Los Angeles, CA, USA).

### 2.5. Metabolic Dysfunction Indices

Metabolic dysfunction indices encompass a range of biomarkers and measurements that highlight irregularities in various metabolic processes, including insulin resistance, lipid metabolism, and the accumulation of body fat. These indices are particularly relevant in identifying conditions such as MetS, which is characterized by a cluster of risk factors that significantly elevate the likelihood of developing cardiovascular diseases and related issues, such as increased arterial stiffness. The calculation of these indices relies on a combination of biochemical assessments and anthropometric measurements, allowing for a comprehensive evaluation of an individual’s metabolic health. The results are expressed through several specific indices, each providing unique insights into metabolic function. These include the atherogenic index of plasma (AIP), which assesses the risk of atherosclerosis; the cardiometabolic index (CMI), which evaluates overall cardiometabolic health; and the lipid accumulation product (LAP), which indicates the risk associated with lipid accumulation. Additionally, the triglycerides to HDL cholesterol ratio (TG/HDL-C) provides insight into lipid profiles, while the triglyceride/glucose index (TyG) serves as a marker for insulin resistance. Further, the TyG-adjusted body mass index (TyG-BMI) and TyG-adjusted waist circumference index (TyG-WC) integrate measurements of body composition and fat distribution, and the visceral adiposity index (VAI) focuses specifically on the amount of visceral fat, which is a key contributor to metabolic disorders. Together, these indices form a critical framework for assessing and understanding metabolic dysfunction and its implications for health [15,16].

The metabolic dysfunction indices were calculated by the following formulas:

AIP = log (TG/HDL-C)                      LAP for females = (WC − 58) × TG (mmol/L)          LAP for males = (WC − 65) × TG (mmol/L)           CMI = (TG/HDL) × WHtR                             VAI for females = WC/(36.58 + (1.89 × BMI)) × TG/0.81 × 1.52/HDL-C VAI for males = WC/(39.68 + (1.88 × BMI)) × TG/1.03 × 1.31/HDL-C  TyG = Ln[TG(mg/dL) × fasting glucose (mg/dL)/2]          TyG-BMI = TyG × BMI                            TyG-WC = TyG × WC                      

### 2.6. Measurement of CAVI

**Figure 2:**
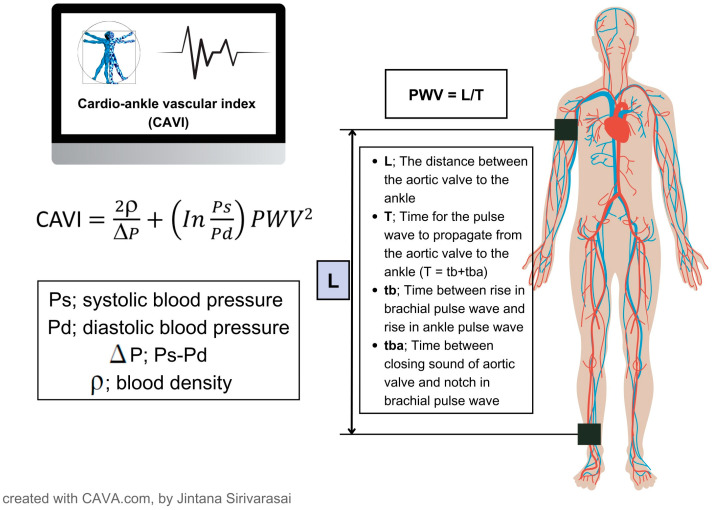
CAVI measurement. Pulse wave velocity (PWV) was obtained by measuring the distance between the aortic valve to the ankle (L), divided by time for the pulse wave to propagate from the aortic valve to the ankle (T). The PWV was then put into the equation for scale conversion. Ps, systolic blood pressure; Pd, diastolic blood pressure; ΔP =Ps − Pd; ρ, blood density; tba, time between rise in brachial pulse wave and rise in ankle pulse wave; tb, time between closing sound of aortic valve and notch in brachial pulse wave.

The CAVI was meticulously assessed in the supine position with concurrent electrocardiogram and phonocardiogram monitoring. The CAVI value was derived using a comprehensive equation that takes into account various physiological parameters (Figure 2). All measurements and calculations were efficiently automated using advanced equipment, specifically the VaSera VS-1000 by Fukuda Denshi, Tokyo, Japan [1]. The mean of the right and left values of CAVI for each participant was used for the analysis. The validity, reproducibility, and blood pressure-independent nature of CAVI have been reported [1]. In this study, we propose CAVI cutoff values of 8.0 and 9.0 (<8 for normal, ≥8 and <9 for borderline, ≥9 for abnormal) [17]. To ensure accurate measurement of CAVI, it is important to provide a peaceful and comfortable environment for participants. It is advisable to encourage them to refrain from smoking, consuming caffeine, or engaging in vigorous exercise for at least 24 h before the test.

### 2.7. Statistical Analysis

Continuous data were represented by mean and standard deviation (SD), while categorical data were presented as percentages. The continuous variables were tested for normality by the Kolmogorov/Smirnov Test before being tested. Homocysteine was natural-logarithmically transformed to normalize its distribution. Statistical analyses included a variance analysis and the Student’s *t*-test for assessing differences in averages, the χ2 test for comparing observed and expected frequencies, and Pearson’s correlation coefficient for evaluating relationships between continuous variables. Hyperhomocysteinemia was classified into moderate (15–30 µmol/L), intermediate (30–100 µmol/L), and severe (>100 µmol/L) [18]. A multiple logistic regression analysis was performed to analyze the association of the CAVI ≥ 9 with each component of MetS, controlling for sex and age. Candidate predictors were selected based on the prior literature and univariate analysis (*p* < 0.2). The evaluation of the post-residual test using Cook’s distance indicated no significant influential observations affecting the model fit, as all values were below 1.0. This finding indicates the reliability of the model estimates. *p* < 0.05 was regarded as statistically significant. The data were analyzed using the Statistical Package for Social Sciences (SPSS) version 23.0 (SPSS Inc., Chicago, IL, USA).

## 3. Results

### 3.1. Comparisons Between Plasma Homocysteine Levels, the Number of Components of MetS, and Clinical, Anthropometric, and Cardiometabolic Parameters

Participants were divided into two groups based on plasma homocysteine (Hcy) levels: low (<15 µmol/L) and high (≥15 µmol/L). The mean Hcy level in the high Hcy group was 20.29 ± 4.63 µmol/L, whereas in the low Hcy group it was 13.25 ± 1.39 µmol/L. These findings align with significant indicators of arterial stiffness, specifically the CAVI, as shown in Table 1. Furthermore, elevated Hcy levels were associated with lower plasma folate and vitamin B12 levels. Significant differences were observed in several parameters between both groups, including body mass index, waist circumference, waist-to-hip ratio, fat mass, and systolic blood pressure (all *p*-values < 0.05). Additionally, the high Hcy group had significantly elevated levels in lipid profile indicators (triglycerides and LDL cholesterol), fasting plasma glucose, and HbA1c compared to the low Hcy group. All cardiometabolic dysfunction indices, including the atherogenic index of plasma (AIP), cardiometabolic index (CMI), lipid accumulation product (LAP), triglycerides/HDL ratio, triglyceride/glucose index (TyG), TyG-adjusted body mass index (TyG-BMI), TyG-adjusted waist circumference (TyG-WC), and visceral adiposity index (VAI), were all significantly higher in the high Hcy group compared to the low Hcy group (all *p*-values < 0.05).

In this study, we explored the relationship between MetS and key parameters associated with arterial stiffness in elderly individuals. Participants were grouped according to the number of MetS components: those with three components (n = 467), four components (n = 269), and five components (n = 25). We aimed to identify statistical differences in both clinical and cardiometabolic indices, as detailed in Table 1. Our findings reveal that participants with four and five MetS components exhibited notably higher levels of BMI, waist circumference, waist-to-hip ratio, systolic blood pressure, triglycerides, fasting plasma glucose, HbA1c, and various cardiometabolic indices, compared to those with three MetS components (all *p*-values < 0.05). Moreover, we observed significant differences in plasma homocysteine (Hcy) levels and the CAVI between participants with three and five MetS components (*p*-values < 0.05).

### 3.2. Association Between the CAVI and Clinical, Anthropometric, and Cardiometabolic Parameters

The findings of our Pearson’s correlation analysis, presented in Table 2, demonstrated the intricate relationship between the CAVI and various clinical parameters. Notably, we found significant positive correlations between CAVI scores and potential factors, such as age, BMI, waist-to-hip ratio, and systolic blood pressure. Furthermore, the CAVI score demonstrated an association with lipid profile, including total cholesterol, triglycerides, and LDL cholesterol, as well as crucial metabolic indicators like fasting plasma glucose and HbA1c, all with *p*-values less than 0.05. In addition to these important correlations, we identified significant alterations in CAVI scores relative to plasma homocysteine levels and indices of cardiometabolic dysfunction: AIP, LAP, TyG, and VAI. The findings clearly indicate that the CAVI is strongly linked to various metabolic and lipid parameters.

### 3.3. Logistic Regression Analysis Between Arterial Stiffness (with a High CAVI; ≥9) and Clinical Variables

**Figure 3:**
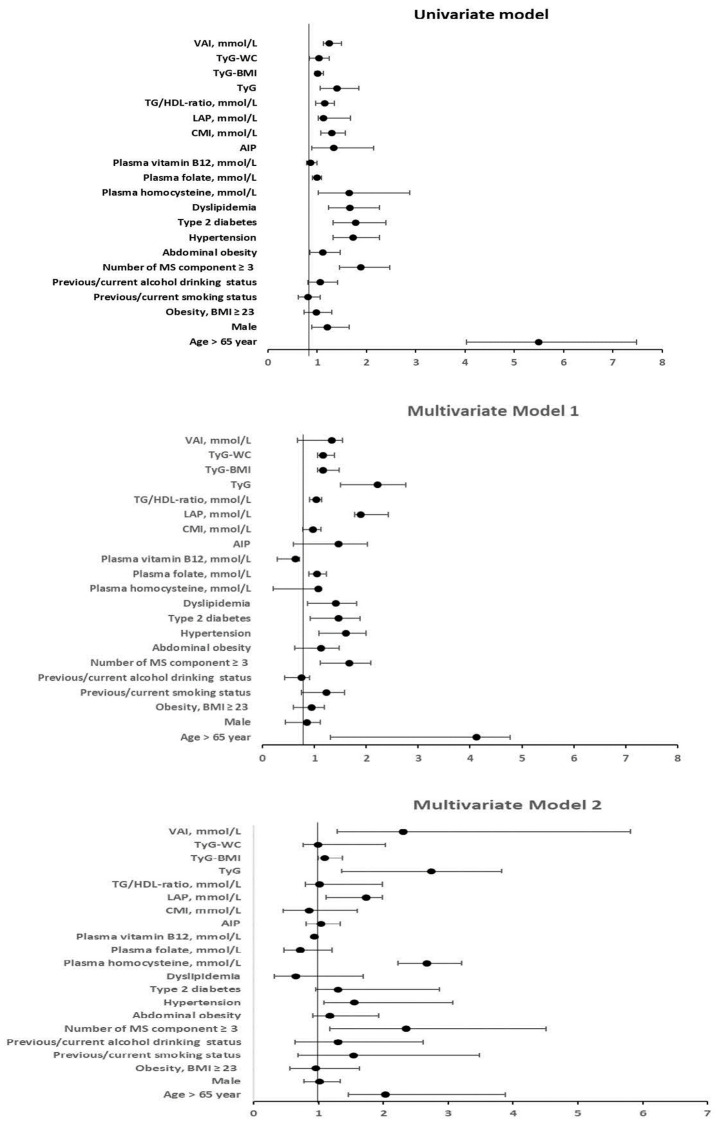
Results of logistic regression analysis between arterial stiffness (with a high CAVI (≥9) and clinical variables. aOR, adjusted odds ratio; CI, confidence interval; OR, odd ratio; model 1 adjusted for age, sex, and smoking and alcohol drinking status; model 2: model 1 adjusted for number of MetS, hypertension, type 2 diabetes, dyslipidemia, plasma homocysteine, and vitamin B12, CMI, LAP, TyG, and VAI; Data are presented as mean ± SD or number (percent), as indicated. AIP: atherogenic index of plasma; BMI: body mass index; CAVI: cardio/ankle vascular index; CMI: cardiometabolic index; DBP: diastolic blood pressure; HDL-C: high-density lipoprotein cholesterol; LAP: lipid accumulation product; LDL-C: low-density lipoprotein cholesterol; SBP: systolic blood pressure; TC: total plasma cholesterol; TG: triglycerides; TG/HDL-C; triglyceride to HDL-C ratio; TyG: triglyceride/glucose index; TyG-BMI: triglyceride-adjusted body mass index; TyG-WC: triglyceride-adjusted waist circumference index; VAI: visceral adiposity index.

A CAVI score of nine or higher is a significant indicator of increased arterial stiffness and associated with adverse cardiovascular outcomes. In this study, we analyzed both univariate and multivariate logistic regression to examine the relationship between high arterial stiffness (CAVI ≥ nine) and various clinical parameters (Figure 3). In the univariate analysis, several significant factors were identified that contribute to an increased risk of arterial stiffness. Notably, individuals over 65 years of age showed a high odds ratio (OR 5.49, 95% CI 4.03–7.48). Furthermore, the presence of more than three components of MetS was associated with an elevated risk (OR 1.89, 95% CI 1.45–2.47), along with hypertension, type 2 diabetes mellitus (T2DM), and dyslipidemia. By conducting a multivariate regression analysis, while adjusting for variables in both models 1 (age, sex, and smoking and alcohol drinking status) and 2 (model 1 adjusted for number of MetS, hypertension, type 2 diabetes, dyslipidemia, plasma homocysteine, and vitamin B12, CMI, LAP, TyG, and VAI), we identified several strong predictors of arterial stiffness risk. These included age over 65 years, having more than three components of MetS, hypertension, and plasma homocysteine. Importantly, we found that increased levels of plasma vitamin B12 may contribute to a reduced risk of arterial stiffness, with odds ratios of 0.64 and 0.94 in models 1 and 2, respectively. Due to the closely association between hyperhomocysteinemia and cardiometabolic dysfunction indices, we combined these parameters to predict the risk of arterial stiffness. We also found that in the univariate analysis, participants with a high level of plasma homocysteine with CMI, LAP, TyG, and VAI showed significant odd ratios for arterial stiffness. The multivariate analysis both model 1 and 2 exhibited strong risk of arterial stiffness based on LAP, TyG, and VAI.

## 4. Discussion

In this study, we have identified a significant association between plasma homocysteine, MetS, and arterial stiffness. Elderly individuals with hyperhomocysteinemia showed significant CAVI scores compared to those with a normal level of Hcy, which underscored a potential predictor of increased arterial stiffness. Previous studies clearly indicated that elevated levels of homocysteine significantly impaired endothelial function by reducing the availability of nitric oxide (NO), resulting in decreased vasodilation and an increase in vascular tone [6]. Furthermore, high homocysteine levels are known to cause substantial endothelial damage through inflammation and leukocyte infiltration. This damage is driven by increased levels of adhesion molecules such as VCAM-1 and ICAM-1 [19]. Additionally, homocysteine unequivocally enhanced cell proliferation in vascular smooth muscle cells in an ROS-dependent manner, which was crucial for maintaining tissue elasticity, wall stress homeostasis, and vessel stiffness [20].

We found that elderly individuals with hyperhomocysteinemia exhibited a significant association with lower plasma levels of vitamin B12 and folate (Table 1). This finding aligns with previous research, which suggested that deficiencies in these vitamins were pivotal in the metabolism of homocysteine. Vitamin B12 and folate serve as essential cofactors in the remethylation pathway of homocysteine to methionine, and their deficiency can disrupt this crucial metabolic process [21]. Hyperhomocysteinemia was found to be less prevalent in the female group. This finding might be supported by a previous study that showed hormone replacement therapy with estradiol decreased blood homocysteine concentrations [22]. This reduction might be related to an increase in the bioavailability of folate and vitamin B12, which are essential cofactors in homocysteine metabolism, as well as promoting the efficient conversion of homocysteine to methionine. These findings highlight the need for targeted dietary interventions or supplementation strategies to improve vitamin status and reduce homocysteine levels in this vulnerable group.

Nutritional deficiencies or excessive high energy intake and chronic inflammation involves immune/inflammation interactions between immune cells and vascular cells; this signal promotes neutrophil activation and lymphocyte suppression. It can lead to a pro-inflammatory state, which is recognized as the underlying mechanism contributing to CVDs [23]. We also observed abnormal levels of plasma Hcy with adiposity indicators, similar to a previous study in elderly populations in Taiwan [24]. Hyperhomocysteinemia potentially exacerbated adipose tissue dysfunction by promoting oxidative stress and increasing the production of pro-inflammatory cytokines, resulting in an impairment of the clearance of Hcy due to a lack of essential B vitamins [25,26]. Furthermore, insulin resistance, often observed in obese individuals, could disrupt the methylation cycle, leading to elevated levels of Hcy [27]. Our analysis suggests that elevated homocysteine (HHcy) levels might contribute to a higher risk of dyslipidemia, specifically manifested by increased triglycerides, decreased HDL-C, and elevated LDL-C. A current study from the National Health and Nutrition Examination Survey (NHANES) also found the correlation between serum homocysteine (Hcy) levels and hyperlipidemia and all-cause mortality of hyperlipidemia patients in a US population [12]. Dyslipidemia in this condition is possibly related to a mechanism by which homocysteine-induced ER stress causes the dysregulation of the endogenous sterol response pathway, leading to increased hepatic biosynthesis and the uptake of cholesterol and triglycerides [28]. In addition, HHcy could affect the down regulation of key players in HDL production (Apo-AI, lecithin-cholesterol acyltransferase; LCAT) and the reduction in liver Apo-AI mRNA expression [29].

In this study, we did not find any significant association between sex-related differences in arterial stiffness and homocysteine metabolism. The underlying physiological mechanisms contributing to sex differences in this context are complex and multifactorial. Hormonal influences and estrogen’s protective role due to estrogen promotes vasoprotective pathways, such as the angiotensin II type 2 receptor (AT2R), which counteracts arterial stiffening [30]. In addition, homocysteine stimulates the secretion of matrix metalloproteinase-12 (MMP12) by macrophages, leading to ECM degradation and arterial stiffening [31]. In men, the absence of estrogen allows homocysteine-induced MMP12 expression to proceed unchecked, leading to greater arterial stiffness. This aligns with findings that men with high homocysteine levels exhibit stronger associations with arterial stiffness than women [31].

For a comprehensive cardiovascular risk assessment, particularly in relation to arterial stiffness, we prioritize indices that combine biochemical profiles and anthropometric measurements. This approach provides a more complete understanding of an individual’s overall cardiometabolic status, particularly concerning plasma homocysteine (Hcy) levels and MetS. The metabolic dysfunction indices from this study showed significantly higher values in the hyperhomocysteinemia group compared to those with normal levels (all *p* values < 0.05). The AIP evaluates the balance between pro-atherogenic and anti-atherogenic lipoproteins, while the LAP combines waist circumference and triglyceride levels, reflecting both the fat distribution and metabolic state—both of which are critically relevant to vascular health [15,16]. Additionally, the TyG index serves as a surrogate marker for insulin resistance, which is associated with arterial stiffness. A previous study highlighted the significance of the TyG index as a reliable indicator for identifying insulin resistance and predicting diabetic kidney disease [32]. In the case of participants with hyperhomocysteinemia, both high fasting plasma glucose and HbA1C levels, which are closely linked to insulin resistance, were identified from our study. A current study on mice also supported this association; homocysteine inhibited pro-insulin receptor cleavage and caused insulin resistance via protein cysteine-homocysteinylation [33].

Based on the interrelated roles of Hcy and MetS in CVDs and vascular dysfunction, we found significantly higher levels of Hcy and CAVI scores in individuals with more MetS components. This finding indicated their roles in vascular stiffness, likely due to its effects on endothelial dysfunction, oxidative stress, and inflammation. High levels of Hcy increase reactive oxygen species, which reduce nitric oxide availability and impair endothelial function [8]. This metabolic problem is worsened by insulin resistance and hypertension in MetS [34]. Hcy also played a significant role in inducing pro-inflammatory gene expression via TF-dependent signaling pathways in monocytes (MCs), leading to MC differentiation and MC-mediated inflammation, thus contributing to vascular inflammation and atherosclerosis [35]. Furthermore, individuals exhibiting multiple components of MetS such as obesity and insulin resistance exhibited a continual state of chronic inflammation, which exacerbated these effects. Components of MetS, such as dyslipidemia and hypertension, accelerated arterial stiffening. Additionally, homocysteine might synergistically promote vascular remodeling through smooth muscle proliferation and extracellular matrix degradation [36].

The increased prevalence of arterial stiffness and its impact on adverse health outcome is an important issue in older populations, especially for early identification and effective interventions. Appropriate surrogate markers, including susceptible factors and biochemical and other clinical measurements, function as significant predictors of CVD consequences. In this study, significant predictors for risk of arterial stiffness from the logistic regression analysis include age > 65 years, number of MetS components, hypertension T2DM, and dyslipidemia and hyperhomocysteinemia status. An epidemiological study reported effects of MetS on arterial function in different age groups. The aging process can contribute to arterial stiffness through several mechanisms, including oxidative stress, endothelial dysfunction, elastin degradation, collagen accumulation, arterial calcification, and chronic inflammation [37].

Understanding the interplay of MetS components provides valuable insights for strategies of arterial stiffness prevention. For instance, addressing insulin resistance and obesity may interact to activate the renin/angiotensin/aldosterone system and the sympathetic nervous system, resulting in reduced vascular tone and stiffness [38]. Physiological and molecular mechanisms of arterial stiffness are complex. Therefore, controlling metabolic dysfunctions like hypertension can lead to the prevention of arterial wall thickening and maintaining compliance, whereas reducing the formation of advanced glycation end products by controlling chronic hyperglycemia is essential to decreasing arterial stiffness [39]. In addition, reducing oxidized LDL levels can prevent foam cell formation, while increasing HDL cholesterol will enhance reverse cholesterol transport, removing excess cholesterol from arterial walls and promoting endothelial function [39]. Both of these strategies lead to reduced inflammation and oxidative stress in the vascular system, contributing to arterial stiffness.

Overall, the results from our study provide significant findings that contribute to our understanding of cardiovascular health in aging populations. The strengths of the study include its focus on an important demographic, the comprehensive assessment of homocysteine and nutritional biomarkers, and integrating Hcy with cardiometabolic markers, leading to potential applications such as identifying high-risk individuals and targeting prevention strategies (e.g., lifestyle, folate/B-vitamin supplementation, metabolic control). However, limitations include potential confounding factors, such as the participants’ overall dietary habits and lifestyle factors, which may not have been fully controlled. This study is focused on the population of Thailand, which may restrict the generalizability of the findings to other demographic groups.

Additionally, the cross-sectional design of the study prevents the establishment of causality between the variables assessed, and the sample size may limit the generalizability of the findings to broader populations. Overall, while the study provides valuable insights, further longitudinal research is necessary to confirm and clarify these associations.

## 5. Conclusions

Our study highlights the important relationship between plasma homocysteine, folate, vitamin B12 levels, and metabolic dysfunction indices in elderly individuals experiencing arterial stiffness. The findings indicated that elevated Hcy levels, along with markers of metabolic dysfunction, are significant predictors of declining vascular health. Moreover, the interaction between vitamin B status and metabolic dysfunction emphasizes the potential of personalized nutrition and promoting accessibility to nutrient-rich foods and supplements for the prevention of vascular complications. In conjunction with these strategies, precision medicine can further enhance treatment by incorporating biomarker-driven risk stratification into clinical practice. This approach allows for tailored early screening and interventions for elderly individuals who are at risk of arterial stiffness. All of these issues also align with global health sustainability goals.

## Figures and Tables

**Table 1:** Characteristics of the participant group according to homocysteine levels and the number of metabolic syndrome components.

Variables	Homocysteine Group		Number of Metabolic Syndrome Components		
	Low (<15 µmol/L) (n = 152)	High (≥15 µmol/L) (n = 732)	3 MetS (n = 467)	4 MetS (n = 269)	5 MetS (n = 25)
Age, years	67.84 ± 4.06	68.82 ± 4.58	68.57 ± 4.46	68.95 ± 4.58	69.12 ± 4.21
Gender, %					
Male	60 (39.5)	607 (82.9)	347 (74.3)	205 (76.2)	21 (84.0)
Female	92 (60.5)	125 (17.1)	120 (25.7)	64 (23.8)	4 (16.0)
Smoking status					
No smoking	124 (81.6)	432 (59.1) *	300 (64.2)	159 (59.1)	16 (64.0)
Ex-smoking	28 (18.4)	300 (40.9)	120 (35.8)	110 (40.9)	9 (36.0)
Alcohol drinking, %					
No drinking	74 (48.7)	208 (28.4) *	154 (38.0)	83 (30.9)	7 (28.0)
Ex-drinking	22 (14.5)	180 (24.6)	105 (22.5)	62 (23.0)	4 (16.0)
Occasional drinking	46 (30.3)	269 (36.7)	172 (36.8)	91 (31.8)	11 (44.0)
Current drinking	10 (6.6)	79 (10.2)	36 (7.1)	33 (12.3)	3 (12.0)
Type 2 diabetes, %	17 (11.3%)	122 (16.7%)	79 (16.9%)	42 (15.6%)	5 (20%)
Dyslipidemia, %	28 (18.4%)	165 (22.6%)	131 (28.1%)	82 (30.5%)	8 (32%)
BMI, kg/m^2^	23.27 ± 3.44	24.72 ± 3.37 *	24.19 ± 3.13	26.29 ± 3.07 ^a^	26.41 ± 1.81 ^a,b^
Waist circumference, cm	85.58 ± 10.18	88.98 ± 10.50 *	87.38 ± 0.42	94.28 ± 7.77 ^a^	94.80 ± 4.71 ^a^
Waist/hip ratio	0.90 ± 0.70	0.93 ± 0.06 *	0.92 ± 0.06	0.95 ± 0.05 ^a^	0.98 ± 0.04 ^a^
Fat mass, %	28.52 ± 7.96	30.77 ± 9.63 *	28.66 ± 8.13	32.26 ± 7.32 ^a^	31.43 ± 6.59
SBP, mm Hg	130.63 ± 19.36	134.44 ± 17.79 *	133.31 ± 17.90	141.03 ± 16.71 ^a^	145.11 ± 12.54 ^a^
DBP, mm Hg	76.49 ± 10.05	77.63 ± 10.09	77.29 ± 9.78	80.27 ± 10.19	81.65 ± 6.90 ^a^
TC, mmol/L	5.32 ± 1.05	5.30 ± 1.16	5.31 ± 1.08	5.18 ± 1.27	5.43 ± 1.05
TG, mmol/L	1.29 ± 0.65	1.43 ± 0.75 *	1.25 ± 0.58	1.77 ± 0.86 ^a^	2.50 ± 0.75 ^a,b^
HDL-C, mmol/L	1.71 ± 0.47	1.49 ± 0.39 *	1.62 ± 0.38	1.27 ± 0.30 ^a^	1.12 ± 0.15 ^a^
LDL-C, mmol/L	3.30 ± 1.01	3.50 ± 1.13 *	3.44 ± 1.04	3.44 ± 1.26	3.80 ± 0.98
Fasting plasma glucose, mmol/L	5.27 ± 0.99	5.47 ± 1.11 *	5.24 ± 0.89	5.90 ± 1.22 ^a^	7.01 ± 1.90 ^a,b^
HbA1C, %	5.90 ± 0.54	5.99 ± 0.71 *	5.86 ± 0.53	6.25 ± 0.84 ^a^	6.77 ± 1.16 ^a,b^
Blood urea nitrogen, mmol/L	4.94 ± 1.69	5.04 ± 1.47	5.37 ± 1.52	5.36 ± 1.39	5.14 ± 1.49
Creatinine, mmol/L	76.71 ± 47.36	80.36 ± 21.26	87.37 ± 23.25	91.01 ± 28.23	88.90 ± 29.32
Homocysteine, µmol/L	13.25 ± 1.39	20.29 ± 4.63 *	18.84 ± 4.85	19.31 ± 5.46	20.98 ± 2.42 ^a,b^
Plasma folate	14.01 ± 8.51	9.09 ± 5.61 *	10.22 ± 6.31	8.68 ± 5.56 ^a^	9.97 ± 5.05
Plasma vitamin B12	921.95 ± 538.14	663.74 ± 377.57 *	713.27 ± 401.72	669.76 ± 436.23	738.38 ± 549.29
CAVI (m/s)	8.75 ± 1.16	9.08 ± 1.11 *	9.01 ± 1.12	9.20 ± 1.23	9.98 ± 0.94 ^a,b^
Metabolic dysfunction indices:					
AIP	0.52 ± 0.27	1.54 ± 0.87 *	0.11 ± 0.08	0.14 ± 0.09 ^a^	3.08 ± 0.17 ^a,b^
CMI, mmol/L	0.79 ± 0.62	0.97 ± 0.89 *	0.72 ± 0.36	1.40 ± 0.95 ^a^	2.14 ± 76 ^a,b^
LAP, mmol/L	32.18 ± 22.69	36.86 ± 23.09 *	29.72 ± 15.01	52.30 ± 24.18 ^a^	75.16 ± 28.43 ^a,b^
TG/HDL-ratio, mmol/L	0.87 ± 0.60	1.09 ± 0.93 *	0.82 ± 0.41	1.56 ± 0.76 ^a^	2.26 ± 0.71 ^a,b^
TyG	8.46 ± 0.49	8.58 ± 0.47 *	8.42 ± 0.36	8.88 ± 0.44 ^a^	9.41 ± 0.43 ^a,b^
TyG-BMI	197.47 ± 39.21	212.43 ± 35.09 *	203.58 ± 30.41	233.94 ± 31.38 ^a^	248.79 ± 21.07 ^a,b^
TyG-WC	727.06 ± 111.69	766.58 ± 100.64 *	740.05 ± 83.54	836.27 ± 75.24 ^a^	894.59 ± 66.77 ^a^
VAI, mmol/L	1.39 ± 0.98	1.48 ± 0.16 *	1.15 ± 0.55	2.15 ± 1.56 ^a^	3.13 ± 1.01 ^a,b^

Data are presented as mean ± SD, or number (percent) as indicated. * Significantly different from low homocysteine group with *p* value < 0.05. ^a, b^ Significantly different from groups of 3 and 4 metabolic syndrome components with *p* values < 0.05. AIP: atherogenic index of plasma; BMI: body mass index; CAVI: cardio/ankle vascular index; CMI: cardiometabolic index; DBP: diastolic blood pressure; HDL-C: high-density lipoprotein cholesterol; LAP: lipid accumulation product; LDL-C: low-density lipoprotein cholesterol; SBP: systolic blood pressure; TC: total plasma cholesterol; TG: triglycerides; TG/HDL-C; triglyceride to HDL-C ratio; TyG: triglyceride/glucose index; TyG-BMI: triglyceride-adjusted body mass index; TyG-WC: triglyceride-adjusted waist circumference index; VAI: visceral adiposity index.

**Table 2:** The partial coefficients of Pearson’s correlation of the CAVI with clinical parameters.

Variable	Correlation Coefficient	*p*-Value
Age, year	0.328	0.000
BMI, kg/m^2^	0.045	0.184
Waist circumference, cm	0.015	0.666
Waist/hip ratio	0.145	0.002
Body fat, %	0.039	0.278
SBP, mm Hg	0.170	0.000
DBP, mm Hg	0.042	0.209
TC, mmol/L	0.131	0.017
TG, mmol/L	0.124	0.005
HDL-C, mmol/L	−0.047	0.165
LDL-C, mmol/L	0.131	0.000
Fasting plasma glucose, mmol/L	0.182	0.000
HbA1C, %	0.158	0.000
BUN, mmol/L	0.097	0.068
Cr, mmol/L	0.098	0.108
Homocysteine, µmol/L	0.545	0.000
Plasma folate, mmol/L	−0.032	0.568
Plasma vitamin B12, mmol/L	0.029	0.396
Metabolic dysfunction indices:		
AIP	0.136	0.000
CMI, mmol/L	0.032	0.339
LAP, mmol/L	0.163	0.001
TG/HDL-ratio, mmol/L	0.065	0.055
TyG	0.139	0.027
TyG-BMI	0.055	0.105
TyG-WC	0.018	0.598
VAI, mmol/L	0.149	0.003

AIP: atherogenic index of plasma; BMI: body mass index; CAVI: cardio/ankle vascular index; CMI: cardiometabolic index; DBP: diastolic blood pressure; HDL-C: high-density lipoprotein cholesterol; LAP: lipid accumulation product; LDL-C: low-density lipoprotein cholesterol; SBP: systolic blood pressure; TC: total plasma cholesterol; TG: triglycerides; TG/HDL-C; triglyceride to HDL-C ratio; TyG: triglyceride/glucose index; TyG-BMI: triglyceride-adjusted body mass index; TyG-WC: triglyceride-adjusted waist circumference index; VAI: visceral adiposity index.

## Data Availability

The data used to support the findings of this study can be made available by the corresponding author upon request.

## Supplementary Materials

The following supporting information can be downloaded at: https://www.mdpi.com/article/10.3390/jcm14092998/s1, Table S1. STROBE Statement—Checklist of Items That Should Be Included in Reports of Cross-Sectional Studies.
