# Supplementary material for: Association Between Plasma Homocysteine, Folate, Vitamin B12 Levels, and Metabolic Dysfunction Indices in Elderly with Arterial Stiffness
Source: J Clin Med. 2025 Apr 26;14(9):2998. doi: 10.3390/jcm14092998 (PMC12072721; doi:10.3390/jcm14092998)
Supplement: Supplementary file 1 [file jcm-14-02998-s001.zip › jcm-3525207-supplementary.pdf]

**Table S1. STROBE Statement—Checklist of Items That Should Be Included in Reports of Cross-Sectional Studies**

| Section/Topic | Item No. | Recommendation | Reported on Page No. |
|---|---|---|---|
| **Title and Abstract** | | | |
| Title | 1 | Indicate the study design with a commonly used term in the title. | Page 1 |
| Abstract | 2 | Provide an informative and structured summary of study objectives, design, setting, participants, variables, results, and conclusions. | Page 1 |
| **Introduction** | | | |
| Background/Rationale | 3 | Explain the scientific background and rationale for the study. | Page 2-3 |
| Objectives | 4 | State specific objectives, including any pre-specified hypotheses. | Page 3 |
| **Methods** | | | |
| Study Design | 5 | Present key elements of the study design. | Page 3 |
| Setting | 6 | Describe the setting, locations, and relevant dates, including periods of recruitment, exposure, follow-up, and data collection. | Page 3 |
| Participants | 7 | State eligibility criteria, sources, and methods of selection of participants. | Page 3 |
| Variables | 8 | Clearly define all outcomes, exposures, predictors, potential confounders, and effect modifiers. | Page 4-6 |
| Data Sources/Measurement | 9 | Provide details on how each variable was measured. If applicable, describe methods used to enhance measurement quality. | Page 4-6 |
| Bias | 10 | Describe efforts to address potential sources of bias. | Page 4-6 |
| Study Size | 11 | Explain how the study size was arrived at. | Page 4-6 |
| Quantitative Variables | 12 | Explain how quantitative variables were handled in the analyses. | Page 4-6 |
| Statistical Methods | 13 | Describe all statistical methods, including those used to control for confounding. | Page 6 |
| **Results** | | | |
| Participants | 14 | Report the number of participants at each stage. | Page 7 |
| Descriptive Data | 15 | Provide demographic, clinical, and social characteristics of the study population. | Page 7-12 |
| Outcome Data | 16 | Summarize key findings for each outcome variable. | Page 7-12 |
| Main Results | 17 | Report effect estimates and confidence intervals, considering confounders. | Page 7-12 |
| Other Analyses | 18 | Provide results of any additional analyses, if applicable. | - |
| **Discussion** | | | |
| Key Results | 19 | Summarize key findings with reference to study objectives. | Page 13-15 |
| Interpretation | 20 | Discuss study results in the context of available evidence. | Page 13-15 |
| Limitations | 21 | Discuss limitations, potential biases, and generalizability. | Page 15 |
| Generalizability | 22 | Discuss the generalizability of study results. | Page 15 |
| **Other Information** | | | |
| Funding | 23 | Disclose funding sources and any conflicts of interest. | Page 16 |
